# Short-Term Nationwide Airport Throughput Prediction With Graph Attention Recurrent Neural Network

**DOI:** 10.3389/frai.2022.884485

**Published:** 2022-06-13

**Authors:** Xinting Zhu, Yu Lin, Yuxin He, Kwok-Leung Tsui, Pak Wai Chan, Lishuai Li

**Affiliations:** ^1^School of Data Science, City University of Hong Kong, Kowloon, Hong Kong SAR, China; ^2^College of Urban Transportation and Logistics, Shenzhen Technology University, Shenzhen, China; ^3^Grado Department of Industrial and Systems Engineering, Virginia Polytechnic Institute and State University, Blacksburg, VA, United States; ^4^Hong Kong Observatory, Kowloon, Hong Kong SAR, China; ^5^Faculty of Aerospace Engineering, Delft University of Technology, Delft, Netherlands

**Keywords:** air traffic network, airport network, throughput prediction, deep learning, graph neural network, complex network

## Abstract

With the dynamic air traffic demand and the constrained capacity resources, accurately predicting airport throughput is essential to ensure the efficiency and resilience of air traffic operations. Many research efforts have been made to predict traffic throughputs or flight delays at an airport or over a network. However, it is still a challenging problem due to the complex spatiotemporal dynamics of the highly interacted air transportation systems. To address this challenge, we propose a novel deep learning model, graph attention neural network stacking with a Long short-term memory unit (GAT-LSTM), to predict the short-term airport throughput over a national air traffic network. LSTM layers are included to extract the temporal correlations in the data, while the graph attention mechanism is used to capture the spatial dependencies. For the graph attention mechanism, two graph modeling methods, airport-based graph and OD-pair graph are explored in this study. We tested the proposed model using real-world air traffic data involving 65 major airports in China over 3 months in 2017 and compared its performance with other state-of-the-art models. Results showed that the temporal pattern was the dominate factor, compared to the spatial pattern, in predicting airport throughputs over an air traffic network. Among the prediction models that we compared, both the proposed model and LSTM performed well on prediction accuracy over the entire network. Better performance of the proposed model was observed when focusing on airports with larger throughputs. We also conducted an analysis on model interpretability. We found that spatiotemporal correlations in the data were learned and shown *via* the model parameters, which helped us to gain insights into the topology and the dynamics of the air traffic network.

## 1. Introduction

Faced with the mismatch between growing air traffic demand and constrained capacity resources, the congestion problem in the air traffic network is expected to remain in a long term. Passenger air travel maintained a year-on-year growth rate of 6–8% from 2010 to 2019 globally before the COVID-19 outbreak (IATA, 2019), while for most of the major airports, there is little opportunity to expand capacity by constructing new infrastructures (Dray, 2020). In addition to capacity constriction, preferred schedule resource is also limited, which may lead to the time displacement (Belobaba et al., 2016; Jacquillat and Odoni, 2018). The unparalleled gap between demand and capacity has caused the air traffic system to be overloaded and leads to extreme flight delays.

Moreover, small deviations between schedules and actual movements can have a disproportionate impact on flight delays, especially during peak hours when the airport operates close to capacity (Jacquillat and Odoni, 2015, 2018). In the empirical study of operations performed on two benchmarking airports in the US and Europe (Odoni et al., 2011), results show that the difference in schedule patterns can affect the states of airport delay. The Newark International airport (EWR) shows a higher average delay and indicates its inability to keep up with the aggressive demand. This inability leads to flight displacement and long delays by being pushed to the later in the day.

The development of better predictions on airport throughput would allow better management of airport operations and alleviate air traffic network congestion. However, it is challenging to accurately predict airport actual throughput due to the highly interacted network effects and complex dynamic mechanisms within the air traffic network. Other airports in the network affect the local airport operations. The propagated influence of upstream delays produced by the sequential flight itinerary, as well as the reflection from downstream anticipated delays due to the collaborative implementation of air traffic control such as the Ground Delay Program, all these situations involve multi-airport spatially in the air traffic system. Besides, dynamic condition changes (e.g., adverse weather, facility limitation or runway configuration in use, etc.), and subjective operation factors like dispatchers or controllers may decide to enhance the arrival throughput to meet the expected arrival demand at the expense of reducing departures, as well as temporal patterns from air traffic characteristics (e.g., seasonal effects, weekly and hourly scheduled properties) will affect the airport actual throughputs.

In this article, we focus on nationwide airport throughput prediction. To address this problem, we propose a deep learning framework named graph attention network stacking with LSTM (GAT-LSTM) for airport departure and arrival throughput prediction, respectively. The proposed model is built and evaluated at the network level and can extract the spatiotemporal correlations in the air traffic network while taking into the topological structure of the airport network. Graph attention network (GAT) is known as the representation of spatial convolution graph neural networks (GNN), which can embed graph-structured traffic features and learn the potential spatial correlations. Then LSTM is adopted to enhance the temporal dependencies extraction within the historical features. We tested our proposed model GAT-LSTM performance on departure and arrival throughput predictions on nationwide airports. Different graph modeling methods are also compared and analyzed. We then discussed the performance for each airport separately and illustrated the model interpretability of the extracted spatial correlations from the graph attention mechanism.

The rest of this article is organized as follows: Section 2 further expounds on the previous studies on the air traffic delays problem. Section 3 introduces the proposed model framework and improved loss function. Section 4 describes the experiment and data. Section 5 further discussed the model performance and illustrates the captured dynamic spatial correlations between airports and the air traffic network. Conclusions and further work are summarized in Section 6.

## 2. Literature Review

We review the relative works about airport traffic network condition prediction and spatiotemporal forecasting methods for road traffic prediction in this section.

### 2.1. Airport Traffic Prediction

Existing research on airport traffic prediction mainly has three kinds of view on building model: microscopic, mesoscopic, and macroscopic (Jacquillat and Odoni, 2015; Simaiakis and Balakrishnan, 2016). Microscopic models consider aircraft individually and adopt simulation tools to reproduce the physical operations of the airport flight, e.g., the Airspace Concept Evaluation System (ACES) of NASA (George et al., 2011) and Future Air Traffic Management Conceptual Environment Tool (FACET) of FAA (Bilimoria et al., 2001). These microscopic models can simulate more realistic operational conditions but are suffered from the computational time consuming and excessive data preparations. Mesoscopic models focus on modeling the runway process and predicting the taxi delay with historical operational data (e.g., pushback time, runway configuration, arrivals, and departures slots, etc.), which is useful for surface operation optimizations (Pujet et al., 1999; Simaiakis and Pyrgiotis, 2010; Simaiakis and Balakrishnan, 2016). While macroscopic models are built based on airport level from the perspective of system planning to analyze the interactions between airports, which coincides with our objective. Macroscopic model methods can be divided into two categories: traditional methods and machine learning or deep learning methods.

Traditional methods focus on using analytical tools to model the mechanism of airport operation, including probabilistic methods (Pathomsiri et al., 2008; Tu et al., 2008), queuing theory methods (Malone, 1995; Hansen, 2002; Pyrgiotis et al., 2013), Bayesian networks models (Xu et al., 2005; Laskey et al., 2012; Rodríguez-Sanz et al., 2019). These models can provide valuable insights into understanding the mechanism of airport operations, however, since the multi-distribution and complex spatiotemporal characteristics within the data (e.g., Long-term temporal repetitive patterns and spatial information from other airports like the downstream airports or other network interactions), these models suffer from poor model performance and have limited capability of feature representation by predefined formulas. Recently, with the development of advanced learning algorithms and the abundant collection of aviation data from multiple sources, machine learning, or deep learning methods show potential for airport traffic prediction problems. These non-parametric approaches do not have well-defined formulas like the traditional analytical tools but can learn samples with complex multi-distributions and have better model performance. A random forest algorithm was adopted to characterize and predict the departure delays in 100 most-delayed origin-destination links in NAS with 19% average test error in classification and 21 min errors in regression (Rebollo and Balakrishnan, 2014). Several machine learning algorithms were applied to the flight on-time performance predictions and compared their performance (Choi et al., 2016). A deep learning methods like recurrent neural network (RNN) are further adopted for flight delay predictions and airport delay predictions (Kim et al., 2016; Zhu and Li, 2021). Deep belief network (DBN) with support vector regression (SVR) was utilized to predict and analyze Beijing International Airport (Yu et al., 2019). This line of research is prevalent recently since machine learning and deep learning models have better prediction accuracy and show superior learning ability to capture useful spatiotemporal correlations within high-dimensional features space, which this study falls into. However, many machine learning and deep learning models usually work as a black box and suffer from model interpretability problems. This situation requires more work to study and analyze the model mechanism where good performance comes from.

### 2.2. Spatiotemporal Forecasting Methods for Road Traffic Prediction

Road traffic prediction is always modeled as time-series forecasting problems, where classical methods, machine learning, and deep learning methods are three typical categories.

Classical methods such as Kalman Filter (Whittaker et al., 1997; Xie et al., 2007), Nonparametric regression (Smith et al., 2002; Clark, 2003), Historical average (Stephanedes et al., 1980), Autoregressive integrated moving average (ARIMA), and its variants (Hamed et al., 1995; Kirby et al., 1997; Williams et al., 1998; Williams, 2001; Kamarianakis and Prastacos, 2005), are developed for years and are mature to learn characteristics of the trend in time series. However, these methods are limited by the linear assumption and inadequate for capturing the large variants by external network effects.

Machine learning methods such as support vector machine (SVM) (Luo et al., 2005; Hong, 2011; Lippi et al., 2013), LASSO (Polson and Sokolov, 2017; Hara et al., 2018), and K-nearest neighbor models (Zhang et al., 2013; Habtemichael and Cetin, 2016) are applied to further improve the performance of traffic volume prediction, but these methods are still limited in mining complex spatial-temporal patterns. Besides, these models require prepared hand-crafted features engineering and additional feature dimension decomposition in advance, which may lose some data properties.

In recent years, deep learning methods achieve remarkable improvement in many fields including traffic prediction, which can extract useful spatiotemporal dependencies directly from raw features. Recurrent neural network (RNN) and its variants long short-term memory unit (LSTM) or gated recurrent unit (GRU) are introduced to process sequential data and show their superior ability in learning the long short-term temporal dependencies (Zhao et al., 2017). Convolutional neural networks (CNN) are first utilized in pattern recognition and image processing while they have been applied in traffic prediction successfully to extract spatial dependencies with Euclidean image-like traffic feature inputs (Tran et al., 2015; Chai et al., 2018). Besides, to be adequate in applying topological features, a Graph neural network (GNN) is introduced (Scarselli et al., 2009), and graph convolutional neural networks (GCN) are further designed (Kipf and Welling, 2016) and applied to traffic prediction problems successfully (Yu et al., 2017; Zhang et al., 2020). As one of the representatives of spatial-domain graph convolution, the Graph attention neural network (GAT) is designed to further extract spatial correlations with its learned attention weights of the links to its adjacent nodes. GAT is proved its learning ability in traffic prediction (Zhang et al., 2018; Guo et al., 2019). Furthermore, to better learn and extract spatial-temporal dependencies within traffic networks, many researchers work on road traffic prediction by combining graph convolutional and recurrent-based methods (Li et al., 2017; Bai et al., 2019; Cui et al., 2019; Guo et al., 2021).

Although there are many existing graph convolutional-based methods applied in road traffic predictions, applications of graph neural network methods are not well explored in air traffic. One aspect is the graph modeling method. Compared to the common sensor location-based road traffic network, how to model the topological graph structure of the airport network is still an open question. Another aspect is model structures, how to build the framework to better capture and illustrate potential spatiotemporal correlations of airport throughputs needs further consideration. To this end, we investigate throughput prediction for airport traffic data to do spatial-temporal modeling with the proposed GAT-LSTM stacked framework to achieve better performance and interpretability.

## 3. Methodology

**Figure 1:**
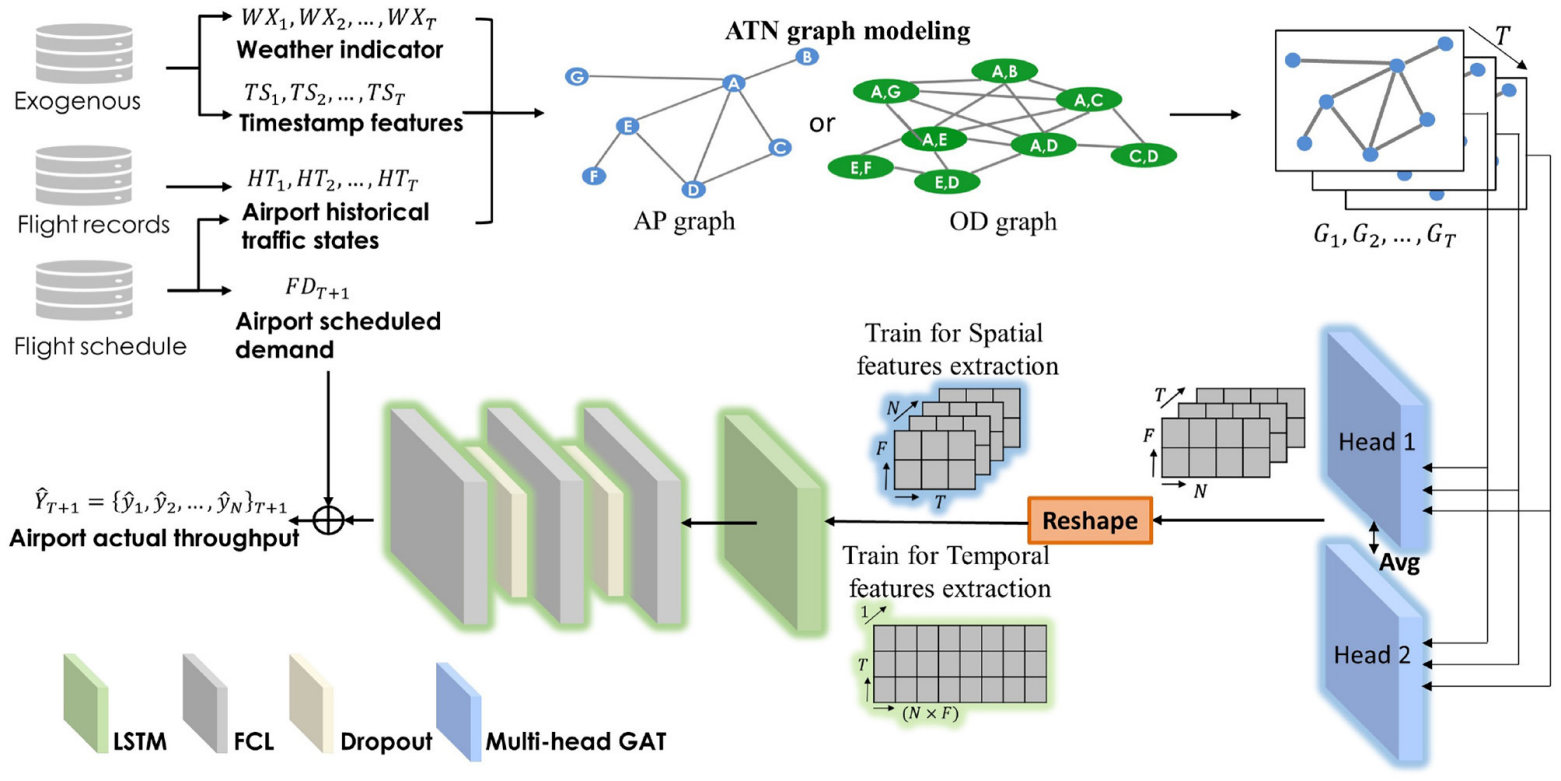
Model structure of GAT-LSTM.

We propose a novel model named graph attention recurrent neural network (GAT-LSTM) to predict the actual airport throughput (arrival and departure) of nationwide airports in the Chinese air traffic network (ATN). This framework first represents the raw ATN traffic data to graph-structured inputs by graph modeling. The raw ATN traffic data include the records on nationwide aircraft movements and flight schedules (departure times and arrival times, origin and destination airports, aircraft types, etc.), which are engineered from Automatic Dependent Surveillance-Broadcast (ADS-B) data source. Then with the timestamp features and weather indicator as exogenous inputs to indicate the scheduled characteristics of air traffic and airport weather condition, respectively, two kinds of ATN graph modeling methods are utilized to build the Airport graph and Origin-Destination graph. GAT is then adopted to extract the dynamic spatial correlations among historical traffic data in ATN airports. Afterward, LSTM is stacked to extract the long-short-term temporal patterns within each airport. Then, a 3-layer fully connected network (FCN) is adopted to do regression for multioutput prediction. In order to better utilize the capability of different layers in the stacked framework, different reshape operations are applied in the training process. With the GAT-LSTM prediction framework, as illustrated in Figure 1, the complex dynamic spatiotemporal correlations of system-wide airports are captured.

Thus, our research problem is defined as learning a function **f**(·) to map previous *T* timestep graph-based air traffic features, weather features and timestamp features *G*_1_, *G*_2_, …*G*_*T*_ gathering with the future demand *FD*_*T*+1_ feature to predict the next timestep actual airport throughputs (for departure and arrival, respectively) for all airports in the ATN, which is formulated as


(1) $$\begin{array}{l} {\hat{\text{Y}}}_{T+1}=\text{f}\left(\left\{G_{1},G_{2},\ldots G_{T}\right\};{FD}_{T+1}\right). \end{array}$$

where ${\hat{\text{Y}}}_{T+1}$ represents the actual departure throughput or arrival throughput of N airports at time *T* + 1, i.e., ${\hat{\text{Y}}}_{T+1}={\left\{{ŷ}_{1},{ŷ}_{2},...,{ŷ}_{N}\right\}}_{T+1}\in {\mathbb{R}}^{N}$, whose element ŷ_*i*_ denote the actual number of aircraft departed from airport *i* or arrived at the airport *i* within the *T* + 1 time interval.

### 3.1. Model Inputs

Model inputs include four categories, weather indicators engineered from exogenous sources to indicate whether the airports are in adverse weather conditions or not, timestamp features as external factors to indicate the scheduled characteristics of air traffic, airport historical traffic features to describe the system-wide flight operation patterns and airport delay states, and future scheduled demand containing the future information on scheduled departure or arrival demand at each airport.

(1) Weather indicator

Weather influences the airport's real-time throughput directly, and in the airport practice, the operations of departure and arrival will be adjusted according to regulations on different weather. Thus, we include a feature *WX*_*t*_ to indicate the weather condition whether it is in visual meteorological conditions (VMC) or instrument meteorological conditions (IMC).


(2) $$\begin{array}{l} {WX}_{t}={\left\{VMC\;or\;IMC\right\}}_{t}. \end{array}$$

(2) Timestamp features

Since the air traffic is a kind of scheduled traffic, it has clear timestamp related patterns, e.g., flights are scheduled to depart at the same o'clock every day or every 2 days constantly; Weekdays and weekends also show different repeat flights schedule patterns, respectively. Timestamp features are adopted to indicate the temporal characteristics of air traffic schedules, including “Time-of-day” and “Day-of-Week.” Due to each timestep being defined as a 15-min interval, the “Time-of-Day” feature is quarter-hourly, i.e., its values range from 0 to 95 per day. As for the Day-of-Week, its values range from 0 to 6. Additionally, to incorporate the cyclical properties of timestamp features, we transform the original values of both features into a sine and cosine representation. At each timestep *t*, the timestamp feature is indicated as


(3) $$\begin{array}{l} {TS}_{t}=\{sin\left(TimeOfDay\right),cos\left(TimeOfDay\right),\\ \;sin\left(DayOfWeek\right),cos\left(DayOfWeek\right){\}}_{t}. \end{array}$$

(3) Historical airport traffic states

Historical airport traffic states are adopted to describe the nationwide air traffic. At each timestep *t*, it can be seen as a snapshot of the air traffic network airport conditions, including Departure delay states (DepDelay), Arrival delay states (ArrDelay), Scheduled departure demand (SDep), Scheduled arrival demand (SArr), Actual departures (ADep), and Actual arrivals (AArr), denoting as


(4) $$\begin{array}{l} {HT}_{t}={\left\{DepDelay,ArrDelay,SDep,SArr,ADep,AArr\right\}}_{t}. \end{array}$$

- *DepDelay*/*ArrDelay DepDelay* ∈ ℝ^*N*^ denotes the average departure delay minutes of each airport within per time interval. Similarly, *ArrDelay* ∈ ℝ^*N*^ is defined as the average arrival delay minutes of each airport within each time interval. These two features are adopted to describe historical flight delay states of the entire ATN accumulated by airports.
- *SDep*/*SArr SDep* ∈ ℝ^*N*^ refers to the number of scheduled departure flights at each airport within one timestep. *SArr* ∈ ℝ^*N*^ refers to the number of scheduled arrival flights correspondingly. They are accounted for by the flight schedules, i.e., scheduled gate-in/gate-out time in the flight itinerary.
- *ADep*/*AArr* With similar definitions, *ADep* ∈ ℝ^*N*^ refers to the number of actual departure flights at each airport within one timestep. *AArr* ∈ ℝ^*N*^ refers to the number of scheduled arrival flights correspondingly. They are engineered from ADS-B data, recording the actual times of aircraft movements to depart and arrive.

(4) Future airport demand

Future airport demand refers to the number of scheduled flights for each airport within the timestep to be predicted *T* + 1. It has the same meaning as *SDep* or *SArr*. This feature is adopted to eliminate the influence of schedule adjustments in air traffic operations, which is engineered from the flight's scheduled departure and arrival information.


(5) $$\begin{array}{l} FD_{T+1}={\left\{x_{1}^{FD},x_{2}^{FD},\ldots ,x_{N}^{FD}\right\}}_{T+1}\in {\mathbb{R}}^{N}. \end{array}$$

### 3.2. ATN Graph Modeling

In theory, a graph is defined as the combinations of nodes (vertices) and edges, wherein the nodes and edges may have attributes, respectively. Air traffic network, similar to road traffic network, has characteristics apart from other networks like citation and social network, i.e., varied traffic states but confirmed physical connectedness structure. Thus, to obey the consistency of graph modeling, we model the ATN graph with a consistent edge and process the traffic state features with nodes. Set *V, E* denotes the node set and edge set, with $X_{t}^{v}$ and $X_{t}^{e}$ as node attributes and edge attributes at timestep *t*, respectively, i.e., $X_{t}^{v}$ includes the time-varied node attributes and $X_{t}^{e}$ represent the varied relationships between each node. Then for each timestep *t*, the ATN graph is modeled as


(6) $$\begin{array}{l} G_{t}=\left(V,E,X_{t}^{v},X_{t}^{e}\right). \end{array}$$

The adjacency matrix is further defined to describe the connectedness of nodes. In the ATN graph, the adjacency matrix is denoted as *A*, wherein its element *A*_*ij*_ = 1 when node *j* is connected to node *i*, otherwise *A*_*i*_*j* = 0. Note that the adjacency matrix also includes self-loop connection, i.e., *A*_*ii*_ = 1. Therefore, the series of graph-structured inputs with previous *T* timesteps are modeled as


(7) $$\begin{array}{l} \left\{G_{1},G_{2},\ldots G_{T}\right\}=\left\{V,E,\left\{X_{1}^{v},X_{2}^{v},\ldots ,X_{T}^{v}\right\},\left\{X_{0}^{e},X_{0}^{e},\ldots ,X_{0}^{e}\right\}\right\}. \end{array}$$

Note that the attributes of edge $\left\{X_{0}^{e},X_{0}^{e},\ldots ,X_{0}^{e}\right\}\in {\mathbb{R}}^{T\times N\times N)}$ are initialized as undirected and unweighted $X_{0}^{e}\in {\mathbb{R}}^{\left(N\times N\right)}$ with all the elements equal to 1 and the correlations within these nodes will be further learned adaptively by the graph attention mechanism of our proposed model. However, in ATN graph modeling the definition of nodes and the edges are worth discussing. In previous studies analyzing the ATN with complex network theory (Cai et al., 2012; Zanin and Lillo, 2013), the common setting of air traffic networks can be defined as two kinds of point-to-point networks. The graph may define the airports as nodes where the edges exist whenever there are flights operated between the two airports, which is called as an Airport graph (APG). While another setting of the ATN can be the Origin-destination graph (ODG), where we set the OD-pairs as the nodes and let edge exist when the two OD-pair involve the same airport, whatever it is origin or destination airport.

Based on the APG definition, the adjacency matrix *A*^*APG*^, wherein its element $A_{ij}^{APG}=1$ if airport *i* and airport *j* have flights operated between, otherwise $A_{ij}^{APG}=0$, and $A_{ii}^{APG}=1$. Besides, at each timestep *t* the set of node attributes is denoted as


(8) $$\begin{array}{l} X_{t}^{{APG}^{v}}=\left\{{HT}_{t}^{v},{WX}_{t}^{v},TS_{t}\right\}. \end{array}$$

**Figure 2:**
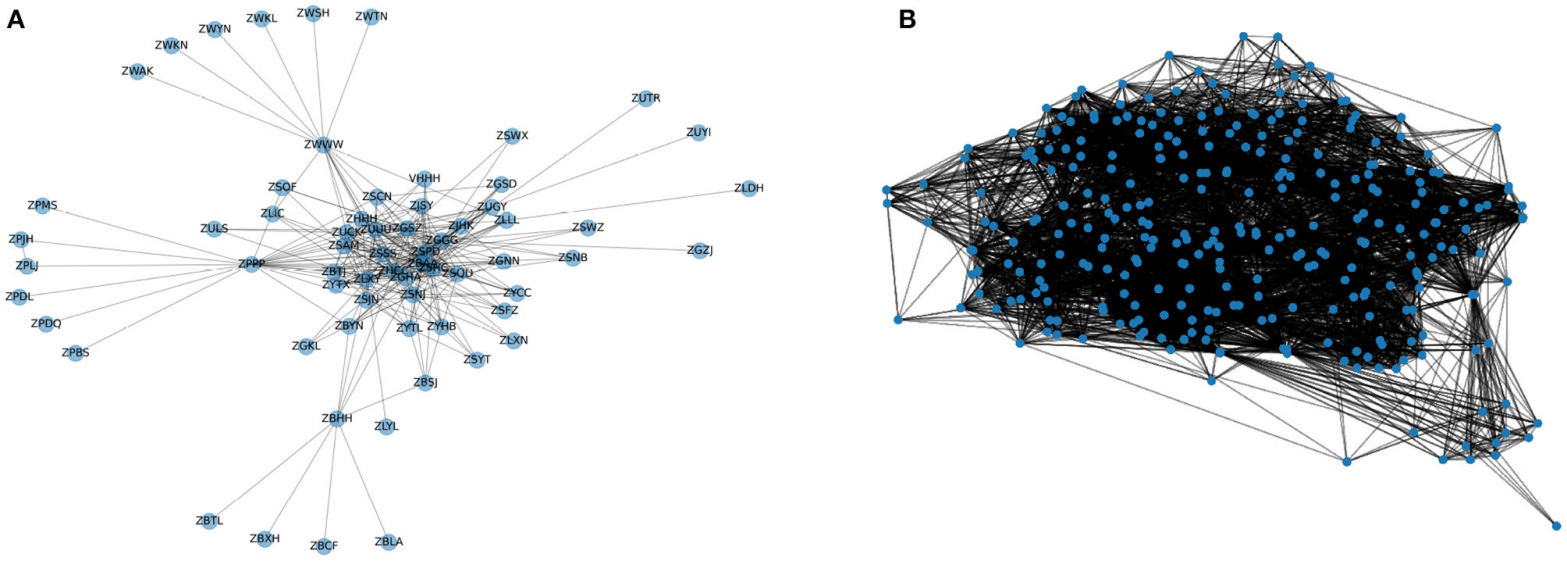
The network structure of two kinds of ATN graph modelings. **(A)** Airport graph (APG). **(B)** OD-pair graph (ODG).

Based on the ODG definition, the element $A_{ij}^{ODG}=1$ if OD-pair *i* and OD-pair *j* involve the same airports in the adjacency matrix *A*^*ODG*^, otherwise $A_{ij}^{ODG}=0$, and also $A_{ii}^{ODG}=1$. Besides, at each timestep *t* the node attributes are formulated as


(9) $$\begin{array}{l} X_{t}^{{ODG}^{v}}=\left\{{HT}_{t}^{O},{HT}_{t}^{D},{WX}_{t}^{O},{WX}_{t}^{D},{TS}_{t}\right\}, \end{array}$$

where *O* refers to the node (i.e., OD-pair) its origin airport, and *D* refers to the destination airport. Apart from the node attributes and adjacency matrix, the other settings of these two graphs keep the same. Figure 2 shows the network structure of the two different graph modeling methods.

As shown in Figure 2, it can be found that APG is a typical small-world network that contains many leaf nodes that only connect to its main airport. It is called a “Hub-and-spoke” structure. As shown in Figure 2, the ZWWW, ZPPP, and ZBHH are three typical regional Hub airports, which are connected to their leaf-airport frequently and have high betweenness centrality.

### 3.3. Prediction Module

The proposed model GAT-LSTM for airport throughput predictions is constructed by a GAT layer with a multi-head mechanism to extract the spatiotemporal correlated dependencies among the network airports. A vanilla LSTM layer is then stacked to extract the temporal patterns within the traffic throughputs series. Then a 3-layer FCN is then utilized to get the final output.

#### 3.3.1. GAT Module

**Figure 3:**
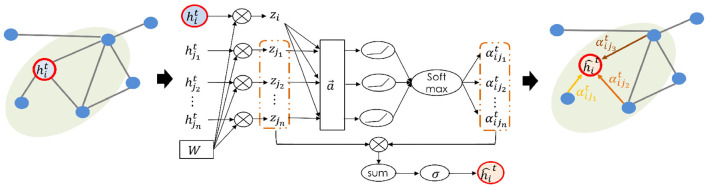
Demonstration of graph attention. Only the first order neighborhood is considered. Note that the learned attributes of edges are bidirectional-weighted.

Set the input of graph attentional layer is ${\text{h}}^{t}=\left\{h_{1}^{t},h_{2}^{t},\ldots ,h_{N}^{t}\right\}$, $h_{i}^{t}\in {\mathbb{R}}^{F}$, where *N* is the number of nodes and *F* is the feature dimension of each node. At the first layer ${\text{h}}^{t}=X_{t}^{v}$, as node attributes in 7. The output of this layer is set as ${\hat{\text{h}}}^{t}=\left\{{ĥ}_{1}^{t},{ĥ}_{2}^{t},\ldots ,{ĥ}_{N}^{t}\right\}$,${ĥ}_{i}^{t}\in {\mathbb{R}}^{F}$, with a new set of node features dimensioned as $\hat{F}$. Then graph attention mechanism is formulated as


(10) $$\begin{array}{l} {ĥ}_{i}^{t}=\sigma \left(\underset{j\in N_{i}}{\sum }\alpha_{ij}^{t}z_{j}\right) \end{array}$$

(11) $$\begin{array}{l} z_{j}=W_{t}h_{j}^{t}, \end{array}$$

where σ is the nonlinear activation function and $W_{t}\in {\mathbb{R}}^{F}$ is a learnable weight matrix for each timestep *t*, as a linear transformation to obtain a more sufficient and higher expression level than original input features. The shared weights are applied to each node. $\alpha_{ij}^{t}$ denotes the learned attention coefficient of node *i* to node *j*. Here, *j* ∈ *N*_*i*_ represents that *j* belongs to the first-order neighborhood of *i* (including *i*), which is predefined by the adjacency matrix. This self-attention mechanism is formulated as


(12) $$\begin{array}{l} \alpha_{ij}^{t}=\frac{\exp\left(\sigma \left(\vec{\text{a}}\left[z_{i}||z_{j}\right]\right)\right)}{\sum_{j\in N_{i}}\exp(\sigma \left(\vec{\text{a}}\left[z_{i}||z_{j}\right]\right)}, \end{array}$$

where α_*ij*_ denotes an alignment function parametrized by a weight vector $\vec{\text{a}}\in {\mathbb{R}}^{2F}$. σ denotes nonlinear function, where applying the LeakyReLU (with slope α = 0.2) in the experiment. ·^⊤^ denotes the matrix transpose and ·||· denotes the concatenation operation. Figure 3 illustrates the demonstration of the graph attention.

Furthermore, to stabilize the learning process of self-attention, the multi-head mechanism (Veličković et al., 2018) is employed. In the experiment, 2 GAT layers are used. These two layers are aggregated. The learned attention weights from these two layers are averaged to obtain the final attention weights.


(13) $$\begin{array}{l} {\hat{\alpha }}_{ij}^{t}=\frac{1}{l}\overset{2}{\underset{l=1}{\sum }}{\alpha_{ij}^{t}}^{\left(l\right)}. \end{array}$$

In GAT, only the first-order neighborhood is considered in the adjacency matrix, which refers to the set of nearest nodes around linked to it, which is suitable for short-term airport throughput prediction in our problem.

#### 3.3.2. Other Regression Modules

After extracting spatial correlations by GAT, a vanilla LSTM is adopted to extract the temporal patterns within its historical air traffic throughput features. LSTM has been widely utilized and shows the superior performance on its long short-term temporal correlation extraction. Its mechanism can be executed via the input gate, the output gate, and the forget gates as introduced by Hochreiter and Schmidhuber (1997), which was further refined by many following application works.

Afterward, the output of LSTM is fed into the following module to predict, where we utilize a 3-layer fully connected network (FCN) to get the final multioutput prediction. Then the future airport demand feature is incorporated to improve the predictions.


(14) $$\begin{array}{l} {\hat{\text{Y}}}_{T+1}=FCN\left(O_{LSTM}\right)+{FD}_{T+1}. \end{array}$$

#### 3.3.3. Training Process

After the input features are reconstructed by the GAT module, the graph-structured output is formulated as


(15) $$\begin{array}{l} \left\{{Ĝ}_{1},{Ĝ}_{2},\ldots {Ĝ}_{T}\right\}=\left\{V,E,\left\{{\hat{X}}_{1}^{v},{\hat{X}}_{2}^{v},\ldots ,{\hat{X}}_{T}^{v}\right\},\left\{{\hat{X}}_{1}^{e},{\hat{X}}_{2}^{e},\ldots ,{\hat{X}}_{T}^{e}\right\}\right\}, \end{array}$$

where the reconstructed node set and edge set are indicated as $\left\{{\hat{X}}_{1}^{v},{\hat{X}}_{2}^{v},\ldots ,{\hat{X}}_{T}^{v}\right\}\in {\mathbb{R}}^{\left(T\times N\times \hat{F}\right)}$ and $\left\{{\hat{X}}_{1}^{e},{\hat{X}}_{2}^{e},\ldots ,{\hat{X}}_{T}^{e}\right\}\in {\mathbb{R}}^{\left(T\times N\times N\times 2\right)}$, respectively.

For node set, as illustrated in the Figure 1, at the training to extract the spatial nodewise correlations, we shape the $\left\{{\hat{X}}_{1}^{v},{\hat{X}}_{2}^{v},\ldots ,{\hat{X}}_{T}^{v}\right\}\in {\mathbb{R}}^{\left(T\times N\times \hat{F}\right)}$ to ${\mathbb{R}}^{\left(N\times T\times \hat{F}\right)}$, while at the training to extract temporal dependencies, it is reshaped to ${\mathbb{R}}^{\left(1\times T\times \left(N\times \hat{F}\right)\right)}$. The idea to separate the training process for different layers aims to better utilize the leaning ability of different modules. In the layer stacking structures, it is difficult to let the different layers perform its specific extraction ability in a one-time united training process. Thus, at the weights training of LSTM, we treated all the nodes not separately in the following temporal extraction with reshaping to ${\mathbb{R}}^{\left(N\times T\times \hat{F}\right)}$ in order to better extract the spatial correlations nodewisely. Then at the temporal features extraction training, we still obey the temporal patterns and concatenate all the nodes together to let LSTM and following layers have a full sight to do the final predictions. Compared to the untied end-to-end training, superior performance and better interpretability are obtained, as shown and discussed in Section 5.3.

For edge set, the responding adjacency matrix with learned attention weights of GAT layers are bidirectional-weighted, indicated as$\left\{{\hat{X}}_{1}^{e},{\hat{X}}_{2}^{e},\ldots ,{\hat{X}}_{T}^{e}\right\}\in {\mathbb{R}}^{\left(T\times N\times N\times 2\right)}$ with each element ${\hat{X}}_{t}^{e}=\left\{\left\{{\hat{\alpha }}_{1j}^{t},{\hat{\alpha }}_{j1}^{t}\right\},\left\{{\hat{\alpha }}_{2j}^{t},{\hat{\alpha }}_{j2}^{t}\right\},\ldots ,\left\{{\hat{\alpha }}_{Nj}^{t},{\hat{\alpha }}_{jN}^{t}\right\}\right\}\in {\mathbb{R}}^{\left(N\times N\times 2\right)}$ at each timestep, where for each node *i* the inflow and outflow weighted from its first-order neighborhood nodes are $\left\{{\hat{\alpha }}_{ij}^{t},{\hat{\alpha }}_{ji}^{t}\right\}$.

The loss function is the standard mean squared error (MSE) between the predicted ŷ_*i*_ and the ground truth *y*_*i*_ ∈ ℝ.


(16) $$\begin{array}{l} Loss\left(\theta \right)={∥{ŷ}_{i}-y_{i}∥}^{2}. \end{array}$$

where θ denotes all the corresponding learnable parameters in the proposed model. At the training process to extract the spatial correlations, θ refers to parameters of GAT layers, and at the training process to extract temporal correlations, θ refers to parameters of other regression modules including LSTM and FCN. The adopted optimization algorithm is Adam (Kingma and Ba, 2015).

## 4. Experiments

### 4.1. Datasets

We collected the raw data from ADS-B data sources. Due to the seasonal a1 July to 30 September, i.e., Quarter 3 in 2017. Therefore, the dataset contains 8,837 timestep samples, that are counted every 15 min to describe the nationwide traffic states. It is noted that only domestic flights are included in the dataset.

**Figure 4:**
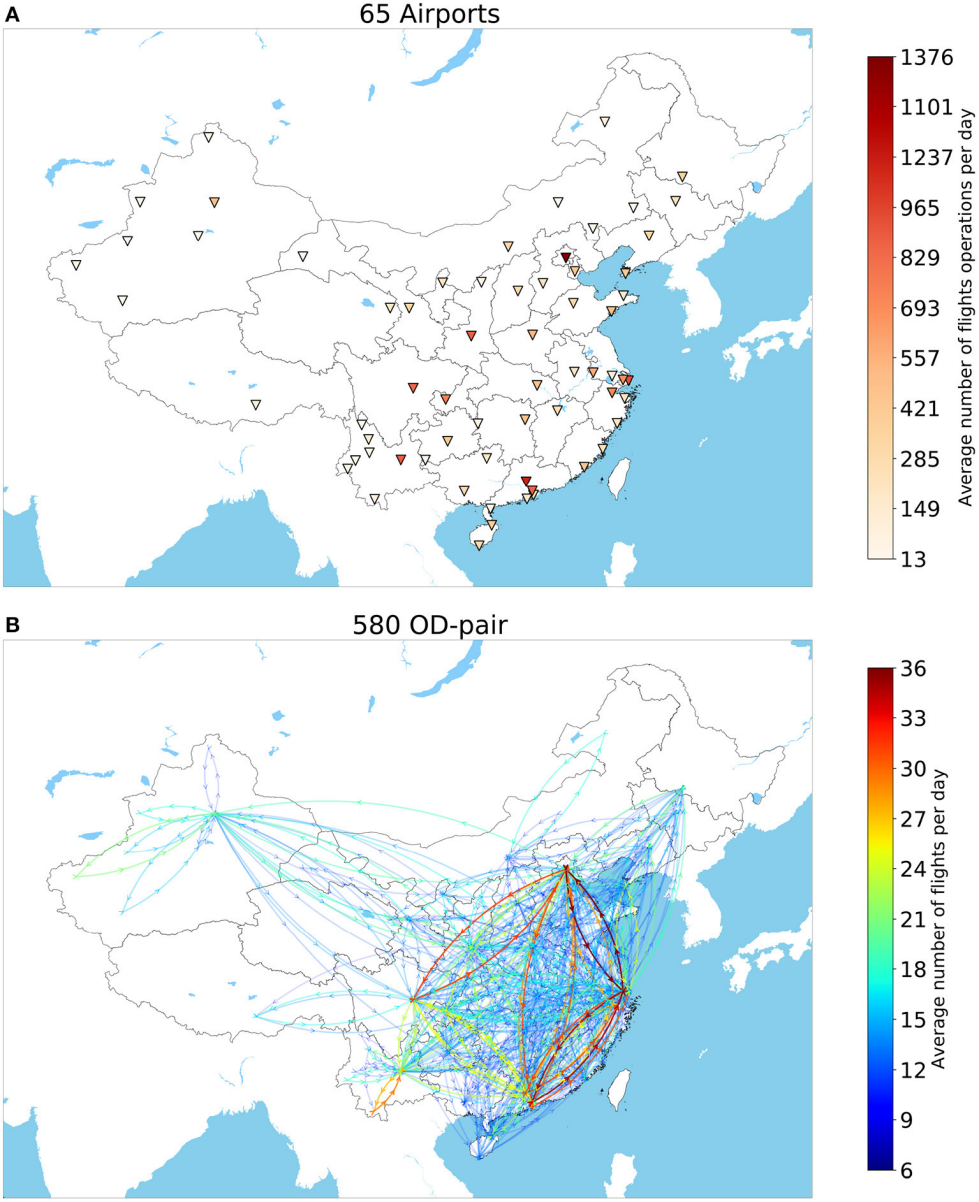
The selected **(A)** 65 airports and **(B)** 580 OD-pair having more than 6 flights per day on average per Chinese aviation network in Quarter 3, 2017. Top: Airports are colored by the total number of flights operations per day including departures and arrivals; Bottom: OD pairs with clockwise directions are colored by the average number of flights operated per day.

Besides, to eliminate the impact of temporary flights and get a stable flight operational network for domestic air traffic, only OD-pairs with more than 6 flights per day on average are included. After data filtering, approximately 73% of flights are included, involving 580 OD-pair and 65 airports. Figure 4 show the simplified Chinese airport network, where the busiest airport Beijing Capital International Airport ZBAA has 1,376 flight operated per day on average while the idlest one is Burqin Kanas Airport ZWKN with 13 flights operated per day on average.

Afterward, training-validation-test sets are split with the sample ratio of around 60%:20%:20% according to time order, i.e., the first 56 days with 56*96 timesteps from 1 July to 25 August is for training, the next 18 days with 18*96 timesteps from 26 August to 12 September is for validation and the rest of 18 days with 18*96 timesteps are used to do testing. Then, all the input features are scaled to get the normal distribution as input with mean and SD of its corresponding values of training data.

### 4.2. Evaluation

#### 4.2.1. Compared Methods

- **Schedule**: Airport scheduled throughputs are counted from the flight scheduled departure time (for departure throughput) and flight scheduled arrival time (for arrival throughput), which works as the baseline.
- **Linear regression**: Linear regression, which is conducted by the one fully connected layer with linear activation function, which indicates the linear mapping between inputs and outputs.
- **Random forest**: Multioutput random forest regression, which is a widely adopted machine learning method in a regression problem.
- **Fully connected network**: Fully connected neural network, which has the same structures as the FCN module in Figure 1.
- **Long Short-Term Memory**: Long Short-Term Memory Network, which has the same structures as in Figure 1 except for the graph attention module. This method focuses on extracting temporal correlations of the inputs, which can be seen as the variant of GAT-LSTM.
- **Graph attention neural network**: Graph attention neural network, which has the same structures in Figure 1 except the LSTM module, which focuses on extracting the spatial correlations of the inputs, which can be seen as the variant of GAT-LSTM.
- **Graph attention neural network stacking with Long short-term memory unit**: Proposed model framework as in Figure 1, stacking GAT and LSTM to extract the spatiotemporal correlations.

#### 4.2.2. Metrics

Two mostly used metrics for multioutput regression are adopted to evaluate model performance, including average Mean Absolute Error (MAE) and average Root Mean Square Error (RMSE). Let $y_{i}^{\left(m\right)}$ and ${ŷ}_{i}^{\left(m\right)}$ represent the actual and predicted throughput of airport *i*, respectively. *N*_*test*_ is the number of samples in the test set and *N* is the number of output dimensions, indicating the *N* airports. The definitions of the two metrics are formulated as:


(17) $$\begin{array}{l} MAE=\frac{1}{N}\overset{N}{\underset{i=1}{\sum }}MAE=\frac{1}{N}\overset{N}{\underset{\left(i=1\right)}{\sum }}\frac{1}{N_{test}}\overset{N_{test}}{\underset{m=1}{\sum }}|y_{i}^{\left(m\right)}-{ŷ}_{i}^{\left(m\right)}| \end{array}$$

(18) $$\begin{array}{l} RMSE=\frac{1}{N}\overset{N}{\underset{i=1}{\sum }}RMSE=\frac{1}{N}\overset{N}{\underset{i=1}{\sum }}\sqrt{\frac{1}{N_{test}}\overset{N_{test}}{\underset{m=1}{\sum }}{\left(y_{i}^{\left(m\right)}-{ŷ}_{i}^{\left(m\right)}\right)}^{2}}. \end{array}$$

### 4.3. Model Performance Over the Network

We compare our proposed model performance with other methods for nationwide 65 airports throughput predictions. We build and train separate models for departure throughput prediction and arrival throughput prediction, respectively. For both departure throughput and arrival throughput predictions, we test the model performances with different lengths of timestep inputs, as shown in Table 1.

**Table 1:** Model performance for departure and arrival throughput predictions.

**Input timesteps**	**1**		**4**		**8**		**12**		**24**	
**Metrics**	**RMSE**	**MAE**	**RMSE**	**MAE**	**RMSE**	**MAE**	**RMSE**	**MAE**	**RMSE**	**MAE**
**(A) DEPARTURE THROUGHPUT (EVALUATE ON 65 AIRPORTS AVERAGE)**										
Schedule	0.99	0.62	-	-	-	-	-	-	-	-
LR	0.99	0.69	0.99	0.69	0.98	0.69	0.98	0.69	0.97	0.69
RF	0.96	0.65	0.93	0.63	0.93	0.63	0.93	0.63	0.92	0.63
FNN	0.82	0.59	0.84	0.60	0.87	0.61	0.88	0.61	0.87	0.61
LSTM	**0.81**	**0.58**	0.80	0.56	0.80	0.56	0.80	0.56	0.80	0.56
GAT^*^	0.82	0.59	0.83	0.59	0.84	0.60	0.87	0.61	0.87	0.62
GAT-LSTM^*^	**0.81**	**0.58**	**0.79**	**0.56**	**0.78**	**0.56**	**0.79**	**0.56**	**0.79**	**0.56**
GAT^†^	1.17	0.67	1.19	0.70	1.14	0.67	1.07	0.65	1.08	0.64
GAT-LSTM^†^	1.17	0.67	1.12	0.67	1.10	0.63	1.10	0.65	1.12	0.66
**(B) ARRIVAL THROUGHPUT (EVALUATE ON 65 AIRPORTS AVERAGE)**										
Schedule	0.93	0.59	-	-	-	-	-	-	-	-
LR	0.83	0.61	0.93	0.69	0.96	0.72	1.37	1.04	1.05	0.84
RF	0.82	0.57	0.81	0.56	0.88	0.65	0.88	0.65	0.88	0.65
FNN	0.80	0.58	0.83	0.59	0.84	0.60	0.88	0.64	0.84	0.60
LSTM	**0.80**	**0.57**	**0.78**	**0.56**	**0.78**	**0.56**	**0.78**	**0.56**	**0.77**	**0.56**
GAT^*^	0.81	0.58	0.83	0.59	0.84	0.59	0.84	0.59	0.84	0.60
GAT-LSTM^*^	0.81	0.58	0.79	0.57	**0.78**	**0.56**	**0.78**	**0.56**	0.78	0.56
GAT^†^	1.15	0.66	1.10	0.66	1.09	0.68	1.07	0.65	1.09	0.64
GAT-LSTM^†^	1.16	0.65	1.06	0.62	1.04	0.61	1.01	0.60	1.01	0.59

* *APG, Airport Graph*.

† *ODG, Origin-Destination Graph*.

*The bold values indicate the lowest prediction errors in each column, for easier comparison*.

Table 1A summarizes the model performance comparison for departure throughput predictions. Results show that GAT-LSTM illustrates the best performance for all the timesteps, followed by the LSTM. Compared to the baseline Schedule, the best performance of GAT-LSTM with 8 timesteps input has about 24.2% improvements in RMSE and 9.7% in MAE. Compared to machine learning methods LR and RF, deep learning methods including FCN, LSTM, GAT, GAT-LSTM, show larger improvements reducing around 21.2%~5.1% RMSE and 21.1%~1.6% MAE. With more timesteps as input, the recurrent based method LSTM shows performance improvements, while for the graph-based method, GAT has worse performance with longer timesteps as inputs increasing from 0.82 to 0.87 RMSE which shows its incapability to capture temporal feature dependency. GAT-LSTM combines the recurrent-based LSTM and graph-based GAT together and has the best performance for all the timesteps, which indicates its capability in capturing the spatiotemporal correlations within features.

For the arrival throughput predictions, as shown in Table 1B, LSTM has the best performance overall with 0.77~0.80 RMSE and 0.56~0.57 MAE while GAT-LSTM shows slightly worse performance with 0.78~0.81 RMSE and 0.56~0.58 MAE, which indicates the importance of temporal features compared with the contribution of topological information to arrival throughput predictions. Compared to Table 1A, results also illustrate that the spatial relations of arrival throughput have smaller dependencies than departure throughputs reflecting the limited or worse improvement in prediction accuracy. This can be interpreted by domain knowledge: Airport arrival operations indeed are less influenced by other airport influences since the dispatchers and pilots' “serve arrival first” style in practice. Thus, arrival throughputs are difficult to be affected by the other airports but are mainly affected by their own temporal pattern. However, local departure operation has more factors that can be reflected by the other airports such as the implementation of a ground delay program as well as the situations of delay propagation by late-arriving flights.

On graph modeling comparisons, it can be shown that for both arrival and departure throughput predictions, the ODG model performance is not good, which indicates the OD graph modeling is not suitable for extracting the spatial correlations for throughput predictions. The reason is from the number of nodes of ODG (580 nodes) is larger than APG (65 nodes) resulting in more computation costs and data consumption. With the limited around 4,800 samples to train, the model by ODG modeling is too big to be trained. Thus, the following results only focus on GAT-LSTM with APG modeling without further notification.

### 4.4. Model Performance in a Single Airport

**Figure 5:**
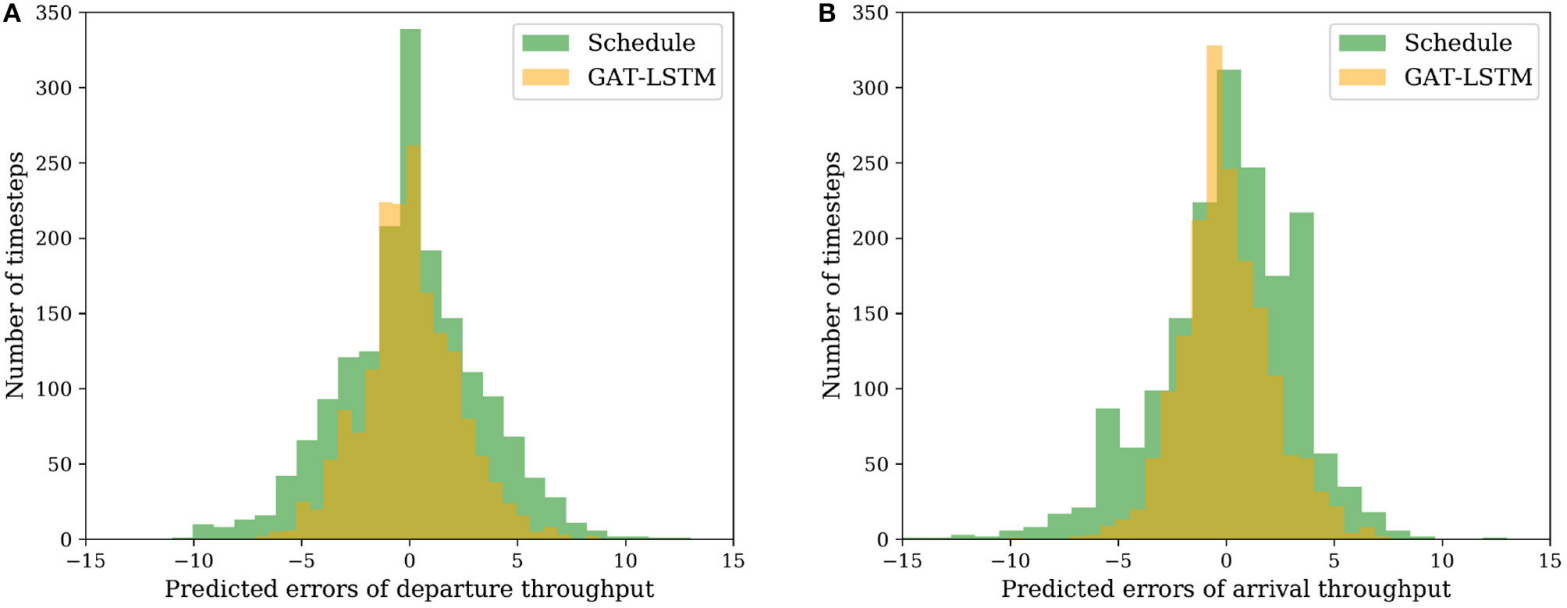
Distributions of predicted errors. **(A)** Departure throughput. **(B)** Arrival throughput.

In the case study, we select the ZBAA as the target airport and evaluate the model performance since it is the busiest airport in the Chinese aviation system. It is noted that the models keep the same with Section 4.4, built and trained for departure and arrival throughput prediction, respectively. Table 2A shows that for departure throughput prediction at ZBAA only, GAT-LSTM has the best performance for all timesteps with around 2.2 RMSE, 1.68 MAE, and 0.66 *R*^2^, which indicates the spatiotemporal correlations prevalent the input features. However, for arrival throughput predictions as shown in Table 2B, LSTM shows better performance, especially with longer timestep inputs i.e., decreasing from 2.22 to 2.06 in RMSE, from 1.69 to 1.57 in MAE, and from 0.83 to 0.85 in *R*^2^. Besides, as shown in Figure 5, compared to the Schedule throughput, the predicted throughput of GAT-LSTM for departure and arrival has a lower variance, as well as more normal distributions with a zero-mean value.

**Table 2:** Model performance of ZBAA departure and arrival throughput predictions.

**Input timesteps**	**1**			**4**			**8**			**12**			**24**		
**Metrics**	**RMSE**	**MAE**	*R* ^2^	**RMSE**	**MAE**	*R* ^2^	**RMSE**	**MAE**	*R* ^2^	**RMSE**	**MAE**	*R* ^2^	**RMSE**	**MAE**	*R* ^2^
**(A) DEPARTURE THROUGHPUT**															
Schedule	3.35	2.50	0.21	-	-	-	-	-	-	-	-	-	-	-	-
LR	3.30	2.50	0.24	3.19	2.44	0.29	3.09	2.38	0.33	3.04	2.35	0.35	2.97	2.29	0.38
RF	3.27	2.48	0.05	2.96	2.28	0.22	2.91	2.19	0.25	3.00	2.30	0.20	2.83	2.18	0.29
FNN	2.35	1.79	0.61	2.41	1.84	0.59	2.35	1.80	0.61	2.38	1.82	0.60	2.33	1.76	0.62
LSTM	2.32	1.78	0.62	2.22	1.69	0.65	2.20	1.67	0.66	2.20	1.68	0.66	2.18	1.67	0.67
GAT	2.31	1.78	0.63	2.27	1.75	0.64	2.27	1.73	0.64	2.58	1.96	0.53	2.45	1.87	0.58
GAT-LSTM	**2.26**	**1.72**	**0.64**	**2.20**	**1.68**	**0.66**	**2.17**	**1.66**	**0.67**	**2.19**	**1.66**	**0.66**	**2.17**	**1.66**	**0.67**
**(B) ARRIVAL THROUGHPUT**															
Schedule	3.16	2.32	0.20	-	-	-	-	-	-	-	-	-	-	-	-
LR	2.60	1.99	0.32	2.68	2.04	0.28	3.08	2.44	0.21	3.11	2.60	0.19	2.32	1.78	0.39
RF	2.35	1.83	0.44	2.30	1.78	0.47	2.30	1.78	0.47	2.27	1.75	0.48	2.27	1.75	0.48
FNN	2.28	1.76	0.82	2.21	1.68	0.83	2.21	1.67	0.83	2.12	1.64	0.84	2.16	1.64	0.84
LSTM	2.22	1.69	0.83	**2.12**	**1.61**	**0.84**	**2.08**	**1.58**	**0.85**	**2.11**	**1.59**	**0.85**	**2.06**	**1.57**	**0.85**
GAT	2.23	1.70	0.83	2.25	1.69	0.82	2.24	1.71	0.83	2.20	1.68	0.83	2.33	1.77	0.81
GAT-LSTM	**2.17**	**1.66**	**0.84**	**2.12**	1.62	**0.84**	**2.08**	1.59	**0.85**	2.13	1.62	0.84	2.07	**1.57**	**0.85**

*The bold values indicate the lowest prediction errors in each column, for easier comparison*.

**Figure 6:**
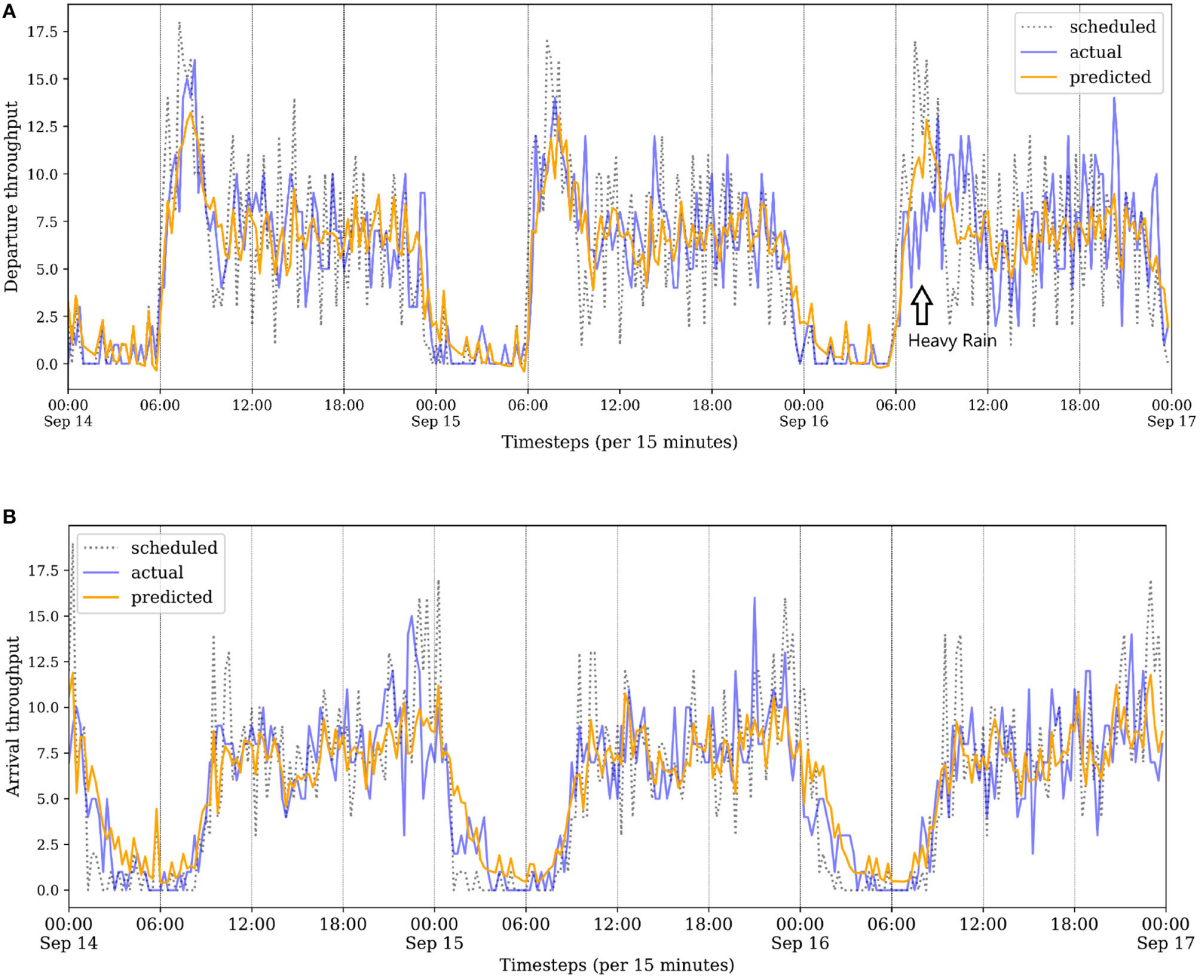
**(A,B)** Throughput visualizations of ZBAA from 14 September to 16 September. ZBAA on 16 September had bad weather in the morning, resulting in actual departure throughput having a big disturbance than schedule from 6:00 to 12:00. Our proposed model GAT-LSTM has better predictions on the situation change.

Figure 6 illustrates three typical days of throughput predictions in the test set. On the third day 16 September, the weather condition is adverse in the morning, which directly influenced the operation of departure flights at ZBAA. Our proposed model GAT-LSTM shows better predictions on this situation change compared to the schedule. Further analysis of captured dynamic spatiotemporal correlations for these days from the perspective of model interpretability is discussed in Section 5.2.2.

## 5. Discussion

### 5.1. Relations Between Performance Improvements With the Airport Scale

**Figure 7:**
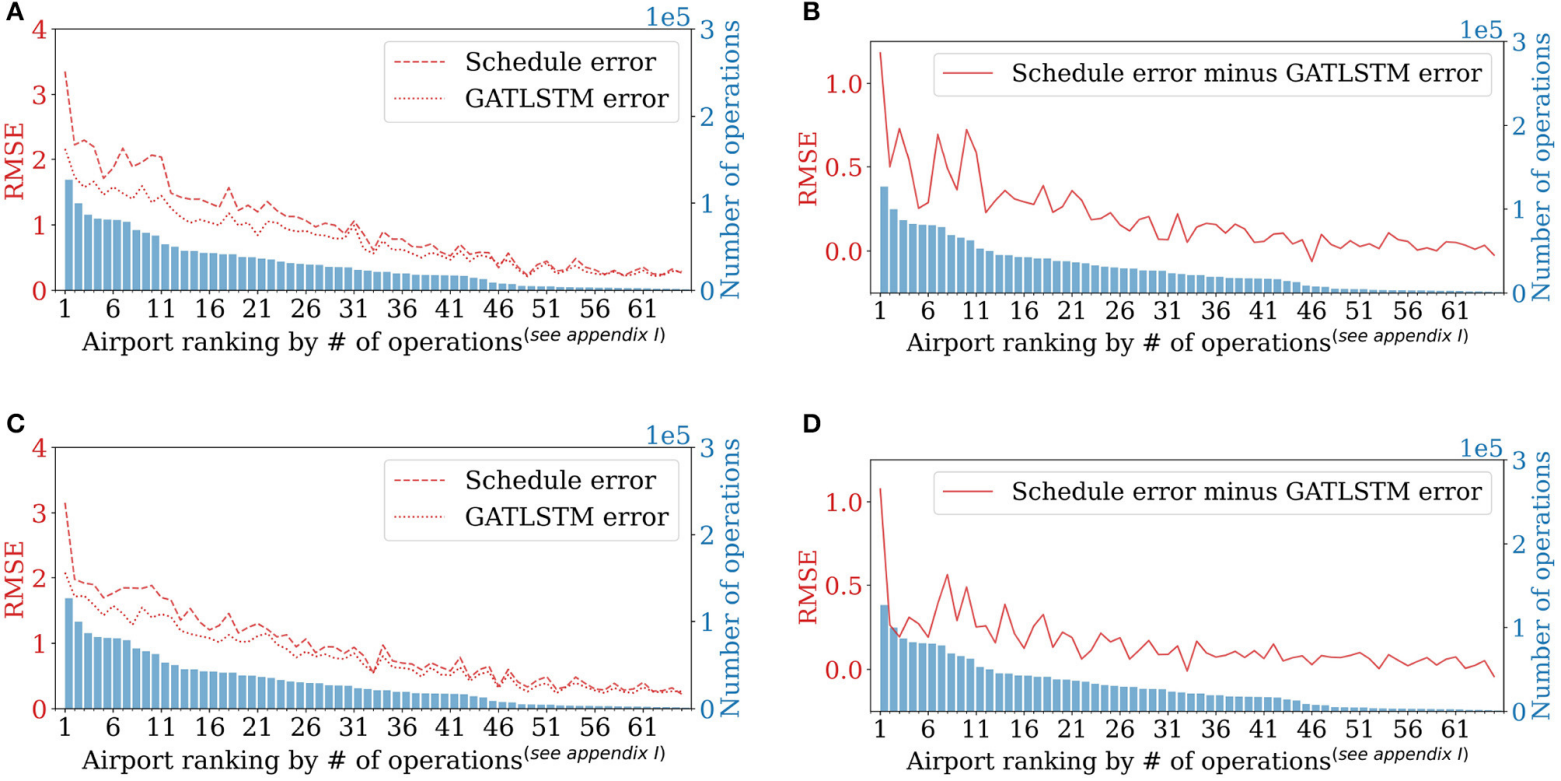
Model performance at each airport. **(A,C)** RMSE of Schedule baseline and GAT-LSTM, respectively. **(B,D)** Reduced RMSE by GAT-LSTM. Airports are sorted by the number of operations including departures and arrivals in Quarter 3, 2017. GATLSTM is with 8 timesteps input

To further analyze model performance for each individual airport, it is found that busier airports are generally more difficult to be predicted accurately compared to less busy airports, however, the proposed model GAT-LSTM can achieve more accuracy improvement (more reduced errors) for busier airports in the network. As shown in Figure 7, we compare the RMSE and reduced RMSE of every single airport for departure throughput and arrival throughput predictions respectively, where airports are sorted from busier to less busy (defined by the total number of flight operations including departures and arrivals in the dataset). For departure throughput prediction and arrival throughput prediction, shown in Figures 7A,C, the prediction RMSE of busier airports is larger than less busy ones in general, which indicates that busier airports are more incapable to operate under schedule, and they are more difficult to be predicted by GAT-LSTM model because of larger operation uncertainties. However, in Figures 7B,D, reduced errors show a decreasing trend from busier airports to less busy airports, indicating busier airports can obtain more accuracy improvement with GAT-LSTM because busier airports have more connections with other airports in the network and they benefit more from the introduced spatiotemporal correlation extraction by GAT-LSTM.

### 5.2. Model Interpretability With APG Modeling

Meaningful topological information and dynamic spatiotemporal correlations can be captured automatically by the proposed GAT-LSTM, which can be reflected by the learned attention weights in the GAT layer. The extracted attention weights ${\hat{X}}_{t}^{e}=\left\{\left\{{\hat{\alpha }}_{1j}^{t},{\hat{\alpha }}_{j1}^{t}\right\},\left\{{\hat{\alpha }}_{2j}^{t},{\hat{\alpha }}_{j2}^{t}\right\},\ldots ,\left\{{\hat{\alpha }}_{Nj}^{t},{\hat{\alpha }}_{jN}^{t}\right\}\right\}$ by GAT layer with APG modeling shows the spatial correlations of different nodes at *t* timestep, indicating the degree of interaction of airports in APG. In the learned weight pair $\left\{{\hat{\alpha }}_{ij}^{t},{\hat{\alpha }}_{ji}^{t}\right\}$, ${\hat{\alpha }}_{ij}^{t},$ indicate the importance of airport *i* to airport *j* at the timestep *t*, and ${\hat{\alpha }}_{ji}^{t},$ indicate the importance of airport *j* to the airport *i* at the timestep *t*. To better illustrate the model interpretability, we select the model GAT-LSTM of departure throughput prediction with input timestep *T* = 8 to show the extracted attention weights.

#### 5.2.1. Spatial Correlations Corresponding to Typical Hub-Spoke Structured Airports

**Figure 8:**
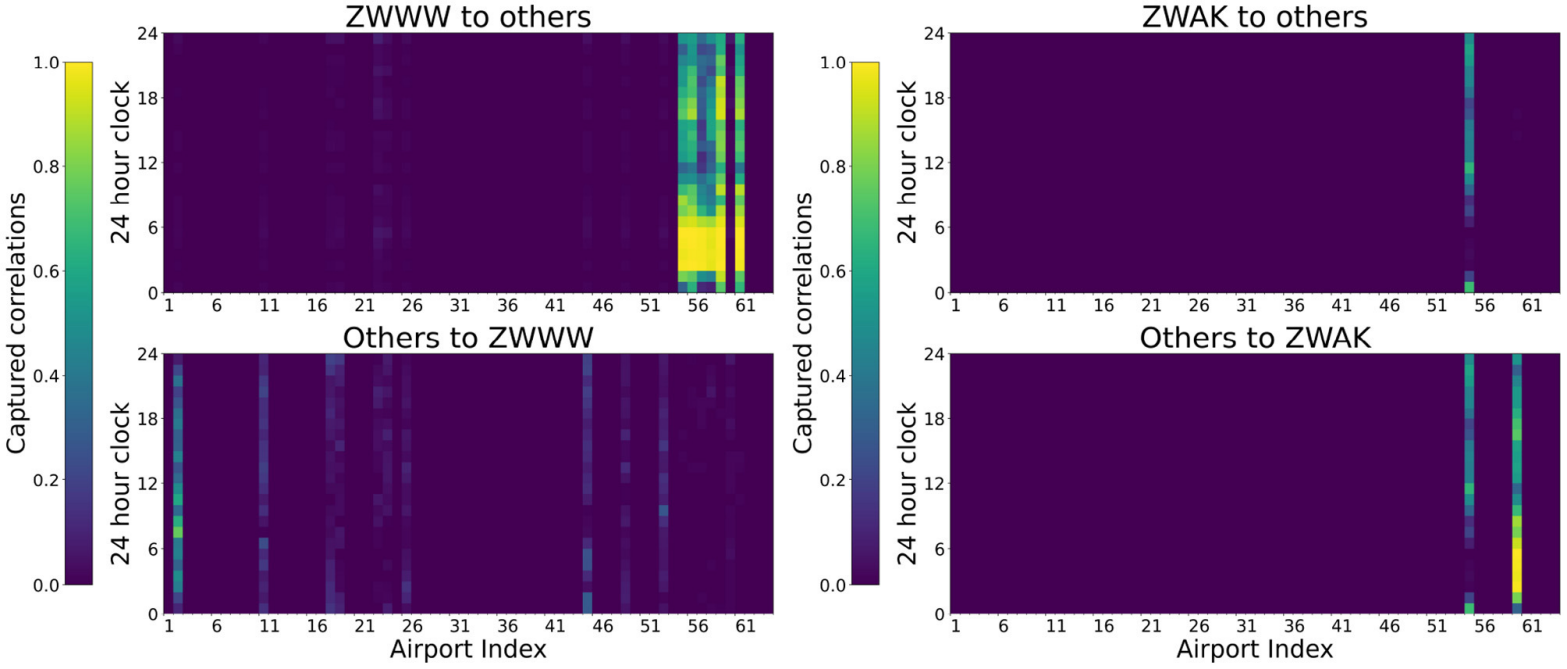
Spatial correlations captured by GAT-LSTM between a hub airport (ZWWW) and one of its spoke airports (ZWAK).

The proposed model GAT-LSTM can capture the specific topological correlations in airport networks. As shown in Figure 2, there is a typical Hub-spoke structured sub-networks in China ATN, which is based on the Urumqi Diwopu International Airport (ZWWW) as the hub. We illustrated the extracted attention weights by the GAT layer of the corresponding hub airport and one of its leaf airports as shown in Figure 8.

As shown in Figure 8, the hub airport ZWWW shows a significant influence on their leaf airports while having a relatively negligible influence on other airports. Besides, the hub airport ZWWW is most influenced by the ZBAA airport who acts a vital role in the entire China ATN. Regarding the temporal correlations during the day, the most influential hours are the morning from 6 a.m. to 9 a.m., while the hour of 7 a.m. shows the biggest influence of ZBAA to ZWWW . For their corresponding spoke airports ZWAK , ZWAK can only influence itself and was greatly impacted by the ZWWW airport most time of the day.

#### 5.2.2. Dynamic Spatial Correlations Corresponding to Typical Days

**Figure 9:**
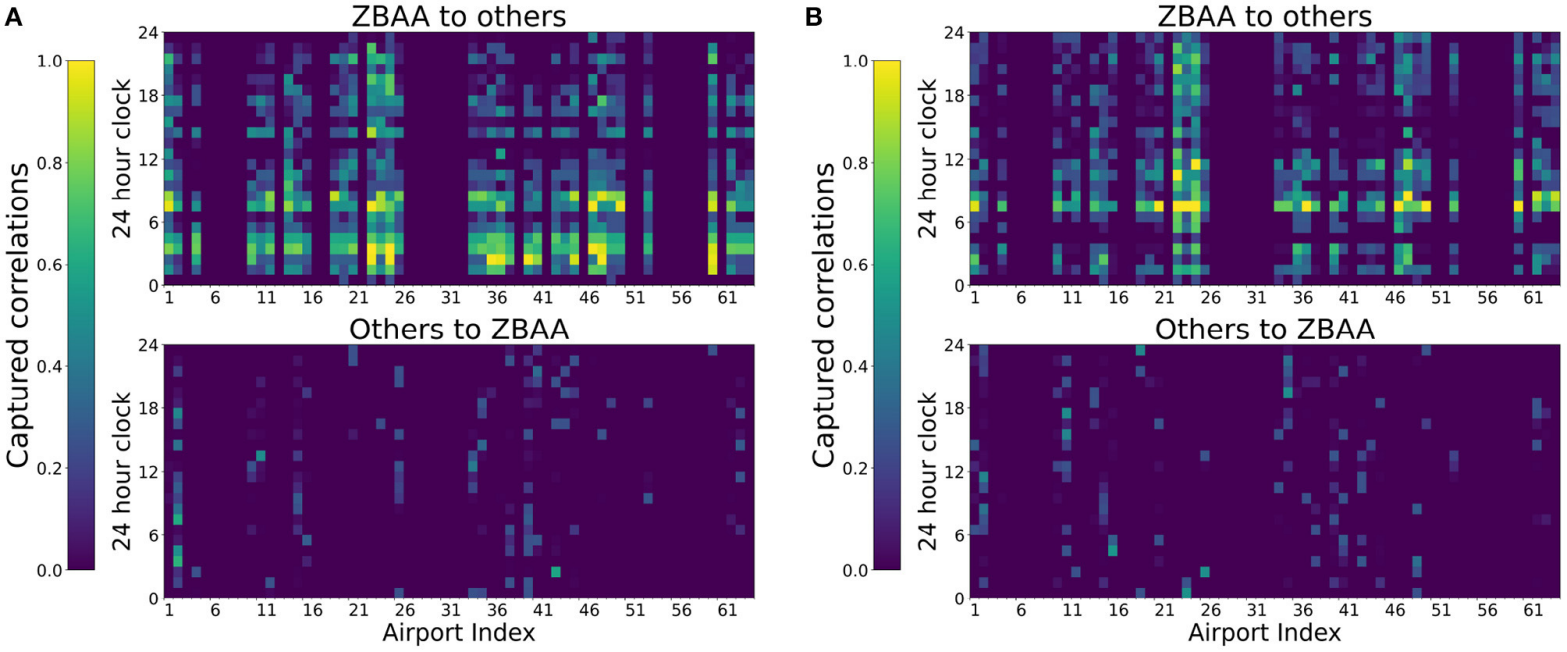
Example of dynamic spatial dependency captured by changes of attention weights: ZBAA. **(A)** ZBAA had bad weather on 16 September morning and **(B)** 15 September is for comparison.

The dynamic spatial dependency can be captured by corresponding attentions weights of the proposed GAT-LSTM. As illustrated in Figure 6A, ZBAA airport on 16 September had bad weather in the morning resulting in abrupt disturbances on actual departures, and 15 September has a relatively normal operation condition. Compared spatial correlations of ZBAA captured by the proposed model between the day of 16 September and the day of the 15th, the spatial dependencies reflected by attention weights show a different pattern as illustrated in Figure 9. On 15 September, the attention weights are more sparse, while the 16 September, the learned weights indicate a more extensive influence on the network-wide airport. This situation also confirms the interacted and dynamic network effect of China ATN.

### 5.3. Separate Training for Different Extraction Modules

**Figure 10:**
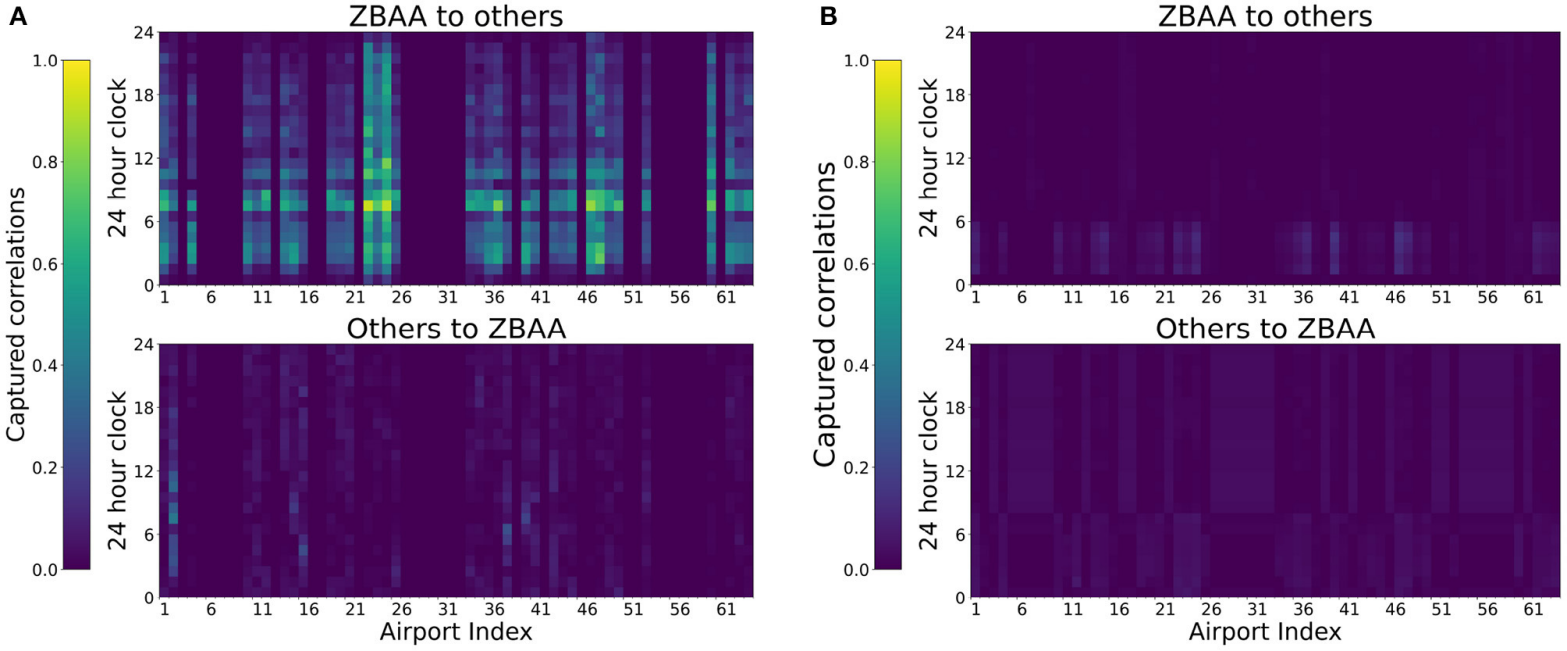
Comparisons of captured spatial correlations between ZBAA and other airports in the different training processes. The attention weights are averaged based on September data. **(A)** With separate training. **(B)** Without separate training.

We adopted separate training processes in the spatial feature extraction by GAT and temporal pattern extraction by LSTM to better utilize their specific learning ability in our proposed layer stacking structures. To extract the corresponding spatial correlations from the graph consisting of various nodes, the normal end-to-end training without obeying the node correlations in the following LSTM and FCN layers was found that the learned attention weights of the GAT layer will be quite smooth and not meaningful, as the comparisons shown in Figure 10. This situation indicates the ineffectiveness of GAT in extracting spatial correlations via the layer stacking structures. The spatial and temporal correlations are extracted by the following layers after GAT layers.

## 6. Conclusion

In this article, we proposed a novel deep learning model framework called GAT-LSTM to predict nationwide 65 airports, actual departure and arrival throughput. Results showed that the proposed model had better performance than baselines methods in departure throughput predictions. While for arrival throughput predictions, the proposed model had a similar model performance as a recurrent-based baseline model LSTM. The results illustrated that temporal dependencies were vital in the predictions, while the departure throughput prediction was more influenced by spatial relations than the arrival throughput predictions. We further explored the model performance by the airport and found that the model had better prediction performance on busier airports than on idler airports.

In the discussion, we illustrated the capability of the graph attention mechanism in revealing spatial correlations in the airport network. The learned attention weights confirmed the effectiveness of the GAT layer in learning the graph-structured spatial correlations. Finally, we explored the impact of training procedures on model performance. Experiments showed that model performance was significantly improved with separate training for each layer. A one-time united training process could not let the different layers perform their specific extraction ability in a layer stacking framework. Future research is needed to develop a training strategy to allow adopted layers to perform their extraction ability.

## Data Availability Statement

The data analyzed in this study was obtained from VariFlight Technology Co. Ltd and The Hong Kong Observatory under Data Confidentiality Agreements. Requests to access these datasets should be directed to LL, lishuai.li@cityu.edu.hk.

## Author Contributions

XZ conceived and carried out the experiments. PC helped with weather data collection and pre-processing. XZ and YL processed and analyzed the data. XZ carried out the experiments in consultation with YL and YH. XZ, YL, and YH contributed to the analysis of the results. XZ and LL contributed to the final version of the manuscript. K-LT and LL provided critical feedback and helped shape the research and analysis. LL supervised the project. All authors contributed to the article and approved the submitted version.

## Funding

The study was supported by the Hong Kong Research Grants Council General Research Fund (Project Nos. 11215119 and 11209717).

## Conflict of Interest

The authors declare that the research was conducted in the absence of any commercial or financial relationships that could be construed as a potential conflict of interest.

## Publisher's Note

All claims expressed in this article are solely those of the authors and do not necessarily represent those of their affiliated organizations, or those of the publisher, the editors and the reviewers. Any product that may be evaluated in this article, or claim that may be made by its manufacturer, is not guaranteed or endorsed by the publisher.
