# Supplementary material for: Short-Term Nationwide Airport Throughput Prediction With Graph Attention Recurrent Neural Network
Source: Front Artif Intell. 2022 Jun 13;5:884485. doi: 10.3389/frai.2022.884485 (PMC9234322; doi:10.3389/frai.2022.884485)
Supplement: Supplementary file 1 [file Table_1.pdf]

APPENDIX I

Table 1 65 airports in Chinese air traffic network.

Airport	Index	Ranking*	Name	City Served	Administrative Division
VHHH	1	21	Hong Kong International Airport	Hong Kong	Hong Kong
ZBAA	2	1	Beijing Capital International Airport	Beijing	Beijing
ZBCF	3	53	Chifeng Yulong Airport	Chifeng	Inner Mongolia
ZBHH	4	27	Hohhot Baita International Airport	Hohhot	Inner Mongolia
ZBLA	5	46	Hulunbuir Hailar Airport	Hailar	Inner Mongolia
ZBSJ	6	33	Shijiazhuang Zhengding International Airport	Shijiazhuang	Hebei
ZBTJ	7	17	Tianjin Binhai International Airport	Tianjin	Tianjin
ZBTL	8	57	Tongliao Airport	Tongliao	Inner Mongolia
ZBXH	9	63	Xilinhot Airport	Xilinhot	Inner Mongolia
ZBYN	10	30	Taiyuan Wusu International Airport Taiyuan	Jinzhong	Shanxi
ZGGG	11	2	Guangzhou Baiyun International Airport	Guangzhou	Guangdong
ZGHA	12	15	Changsha Huanghua International Airport	Changsha	Hunan
ZGKL	13	41	Guilin Liangjiang International Airport	Guilin	Guangxi
ZGNN	14	29	Nanning Wuxu International Airport	Nanning	Guangxi
ZGSD	15	39	Zhuhai Jinwan Airport	Zhuhai	Guangdong
ZGSZ	16	6	Shenzhen Bao'an International Airport	Shenzhen	Guangdong
ZGZJ	17	49	Zhanjiang Airport	Zhanjiang	Guangdong
ZHCC	18	12	Zhengzhou Xinzheng International Airport	Zhengzhou	Henan
ZHHH	19	16	Wuhan Tianhe International Airport	Wuhan	Hubei
ZJHK	20	22	Haikou Meilan International Airport	Haikou	Hainan
ZJSY	21	28	Sanya Phoenix International Airport	Sanya	Hainan
ZLDH	22	59	Dunhuang Mogao International Airport	Dunhuang	Gansu
ZLIC	23	35	Yinchuan Hedong International Airport	Yinchuan	Ningxia
ZLLL	24	25	Lanzhou Zhongchuan International Airport	Lanzhou	Gansu
ZLXN	25	40	Xining Caojiabao Airport	Xining, Haidong	Qinghai
ZLXY	26	5	Xián Xianyang International Airport	Xián, Xianyang	Shaanxi
ZLYL	27	50	Yulin Yuyang Airport	Yulin	Shaanxi
ZPBS	28	64	Baoshan Yunrui Airport	Baoshan	Yunnan
ZPDL	29	52	Dali Airport	Dali	Yunnan
ZPDQ	30	62	Diqing Shangri-La Airport	Shangri-La	Yunnan
ZPJH	31	47	Xishuangbanna Gasa International Airport	Jinghong	Yunnan
ZPLJ	32	43	Lijiang Sanyi International Airport	Lijiang	Yunnan
ZPMS	33	54	Dehong Mangshi Airport	Dehong	Yunnan
ZPPP	34	4	Kunming Changshui International Airport	Kunming	Yunnan
ZSAM	35	18	Xiamen Gaoqi International Airport	Xiamen	Fujian
ZSCN	36	31	Nanchang Changbei International Airport	Nanchang	Jiangxi
ZSFZ	37	32	Fuzhou Changle International Airport	Fuzhou	Fujian
ZSHC	38	9	Hangzhou Xiaoshan International Airport	Hangzhou	Zhejiang
ZSJN	39	26	Jinan Yaoqiang International Airport	Jinan	Shandong
ZSNB	40	37	Ningbo Lishe International Airport	Ningbo	Zhejiang

Table 1 – continued from previous page

Airport	Index	Ranking*	Name	City Served	Administrative Division
ZSNJ	41	11	Nanjing Lukou International Airport	Nanjing	Jiangsu
ZSOF	42	36	Hefei Xinqiao International Airport	Hefei	Anhui
ZSPD	43	3	Shanghai Pudong International Airport	Shanghai	Shanghai
ZSQD	44	13	Qingdao Liuting International Airport	Qingdao	Shandong
ZSSS	45	10	Shanghai Hongqiao International Airport	Shanghai	Shanghai
ZSWX	46	44	Sunan Shuofang International Airport	Wuxi, Suzhou	Jiangsu
ZSWZ	47	38	Wenzhou Yongqiang International Airport	Wenzhou	Zhejiang
ZSYT	48	42	Yantai Penglai International Airport	Yantai	Shandong
ZUCK	49	8	Chongqing Jiangbei International Airport	Chongqing	Chongqing
ZUGY	50	20	Guiyang Longdongbao International Airport	Guiyang	Guizhou
ZULS	51	45	Lhasa Gonggar Airport	Lhasa,Shannan	Tibet
ZUTR	52	48	Tongren Fenghuang Airport	Tongren	Guizhou
ZUUU	53	7	Chengdu Shuangliu International Airport	Chengdu	Sichuan
ZUYI	54	60	Xingyi Wanfenglin Airport	Xingyi	Guizhou
ZWAK	55	58	Aksu Airport	Aksu	Xinjiang
ZWKL	56	56	Korla Airport	Korla	Xinjiang
ZWKN	57	65	Burqin Kanas Airport	Burqin	Xinjiang
ZWSH	58	51	Kashgar Airport	Kashi	Xinjiang
ZWTN	59	61	Hotan Airport	Hotan	Xinjiang
ZWWW	60	14	Urumqi Diwopu International Airport	Urumqi]	Xinjiang
ZWYN	61	55	Yining Airport	Yining	Xinjiang
ZYCC	62	34	Changchun Longjia International Airport	Changchun	Jilin
ZYHB	63	23	Harbin Taiping International Airport	Harbin	Heilongjiang
ZYTL	64	19	Dalian Zhoushuizi International Airport	Dalian	Liaoning
ZYTX	65	24	Dalian Zhoushuizi International Airport	Dalian	Liaoning

* Airport Ranking is ordered by the number of total operations including departures and arrivals in Quarter 3, 2017.
